# ALD-Deposited Hydroxyl-Rich NiO_x_ to Enhance SAM Anchoring for Stable and Efficient Perovskite Solar Cells

**DOI:** 10.3390/molecules30061299

**Published:** 2025-03-13

**Authors:** Fengming Guo, Xuteng Yu, Yuheng Li, Yong Chen, Chi Li, Chunming Liu, Peng Gao

**Affiliations:** 1 College of Chemistry and Materials Science, Fujian Normal University, Fuzhou 350108, China; 2 State Key Laboratory of Structural Chemistry, Fujian Institute of Research on the Structure of Matter, Chinese Academy of Sciences, Fuzhou 350002, China; 3 Laboratory for Advanced Functional Materials, Xiamen Institute of Rare Earth Materials, Haixi Institute, Chinese Academy of Sciences, Xiamen 361021, China; 4 Fujian College, University of Chinese Academy of Sciences, Fuzhou 350002, China; 5 University of Chinese Academy of Sciences, Beijing 100049, China

**Keywords:** hybrid hole selective layer, NiO_x_, energy-level alignment, passivation, inverted perovskite solar cells

## Abstract

The interface between nickel oxide (NiO_x_) and self-assembled monolayers (SAMs) in perovskite solar cells (PSCs) often suffers from limited adsorption strength, poor energy-level alignment, and inadequate defect passivation, which hinder device performance and stability. To address these issues, we introduce a hybrid hole selective layer (HSL) combining atomic layer deposition (ALD)-fabricated NiO_x_ with full-aromatic SAM molecules, creating a highly stable and efficient interface. ALD NiO_x_, enriched with hydroxyl groups, provides robust adsorption sites for the SAM molecule MeO-PhPACz, ensuring a strong, stable interaction. This hybrid HSL enhances energy-level alignment, hole selectivity, and defect passivation at the NiO_x_/perovskite interface. Devices utilizing this approach demonstrate significant performance improvements, achieving a power conversion efficiency (PCE) of 21.74%, with reduced voltage losses and minimal hysteresis. Furthermore, operational stability tests reveal enhanced durability under elevated humidity and temperature conditions. These findings highlight the potential of ALD NiO_x_ and SAM hybrid HSL to overcome existing barriers, advancing the commercial viability of PSC technologies.

## 1. Introduction

Organic–inorganic hybrid perovskite solar cells (PSCs) have made remarkable strides in power conversion efficiency (PCE) in a short time [1,2,3,4,5]. Among these, the inverted p-i-n structure has attracted considerable attention for its low-temperature fabrication process, reduced hysteresis, enhanced stability, and ability to meet the structural demands of tandem solar cells [6,7]. In the fabrication of inverted PSCs, a hole selective layer (HSL) is first deposited onto the indium tin oxide (ITO) substrate. This layer not only ensures efficient hole collection but also minimizes charge recombination at the interface, which plays a critical role in the crystallization and nucleation of the perovskite material. Therefore, the development of high-performance HSL is essential for achieving high-efficiency inverted PSCs.

At present, a wide variety of HSLs have been employed in inverted PSCs. Among these, nickel oxide (NiO_x_) has garnered significant attention owing to its cost-effectiveness, facile manufacturability, outstanding hole extraction efficiency, and well-aligned energy levels [8,9,10,11,12,13]. The performance and stability of NiO_x_-based PSCs are frequently hindered by undesirable reactions between the perovskite layer and highly reactive Ni^3+^ species, as well as charge carrier recombination at the NiO_x_/perovskite interface [14,15,16]. To mitigate these issues, the combination of NiO_x_ with self-assembled monolayers (SAMs) has emerged as a highly effective strategy [9,12,13,17,18]. Wang et al. employed bromobenzoic acid (Br-BA) to modify the surface of NiO_x_ nanoparticles, reducing interfacial defects and trap states, enhancing perovskite crystallization quality, and optimizing energy-level alignment [19]. Yu et al. treated NiO_x_ nanoparticles with H_2_O_2_, which enhanced the film’s conductivity and surface hydroxyl content, promoting SAM anchoring [20]. Zhao et al. incorporated sodium hexametaphosphate (SHMP) to modify NiO_x_, improving the surface hydroxyl density and film uniformity, thereby enhancing the anchoring of Me-4PACz and reducing the open-circuit voltage loss in wide bandgap perovskite solar cells [13].

In a previous work, we proposed a Ni vacancy defect modulate approach to leverage the conformal growth and surface self-limiting reaction characteristics of atomic layer deposition (ALD)-fabricated NiO_x_ by varying the O_2_ plasma injection time to induce self-doping [21]. We fabricated ultrathin NiO_x_ films with excellent conductivity, an optimal Ni^3+^/Ni^2+^ ratio, and stable surface states, serving as the HSL in p-i-n PSCs. However, interface defects at the NiO_x_/PVK interface can induce perovskite degradation, and a higher density of defects is typically present at the bottom perovskite interface. Building upon prior studies, the incorporation of SAMs with NiO_x_ has been demonstrated to enhance the surface hydroxyl density, strengthen SAM anchoring, mitigate surface defects, and ultimately improve the film quality [13,19,20]. Consequently, we propose the introduction of a novel SAM at the NiO_x_/PVK interface to address the aforementioned detrimental characteristics.

Herein, we deposited a layer of MeO-PhPACz (hereafter referred to as MeO-Ph) [22] onto the ALD-deposited NiO_x_, which has a surface rich in -OH groups, thereby providing more adsorption sites for the SAM molecules. Compared to ITO/MeO-Ph, the binding energy between ALD-NiO_x_ and MeO-Ph is increased, with stronger and more stable interactions and also improved hole extraction capability. In contrast to ALD-NiO_x_, MeO-Ph effectively passivates the surface defects of NiO_x_, significantly suppressing redox reactions at the HSL/PVK interface and notably enhancing the work function of the HSL. As a result, the hybrid HSL with well-aligned energy levels, stable surface states, and efficient hole extraction was achieved. The corresponding device showed a marked increase in PCE from 19.86% (ALD-NiO_x_) and 20.53% (MeO-Ph) to 21.74% (ALD-NiO_x_/MeO-Ph), with minimal hysteresis. Stability tests conducted on unencapsulated devices under various aging conditions demonstrated that devices based on ALD-NiO_x_/MeO-Ph exhibited excellent long-term stability.

## 2. Results and Discussion

**Figure 1:**
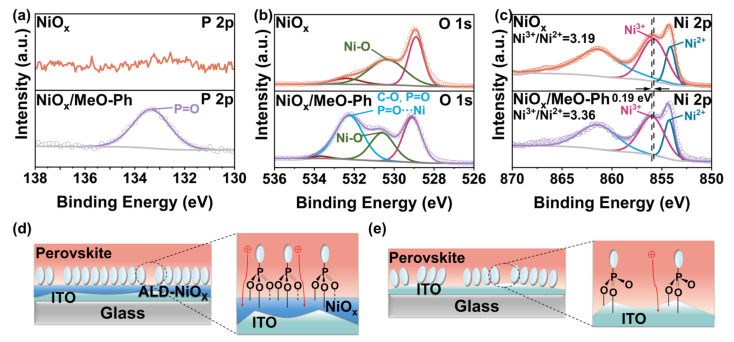
XPS spectra of (**a**) P 2p, (**b**) O 1s, and (**c**) Ni 2p for the three films, and adsorption patterns of MeO-Ph on (**d**) NiO_x_ and (**e**) ITO surfaces.

Excellent optical transmittance significantly influences the short-circuit current of the device. We measured the transmittance of three thin films in the 350 nm–800 nm wavelength range. The results show that all three films exhibit transmittance above 90%, with MeO-Ph achieving an optical transmittance exceeding 95% due to its ultrathin nature. To investigate the interaction between MeO-Ph and NiO_x_ (unless otherwise specified, NiO_x_ refers to ALD-NiO_x_), we used XPS to evaluate the surface chemical properties of ITO/NiO_x_ before and after MeO-Ph adsorption. Pure MeO-Ph adsorbed on an ITO substrate was used as a reference sample, with the elemental composition summarized in Table S1. The differences in phosphorus (P) in Figure 1a and nitrogen (N) in Table S1 confirm the successful adsorption of MeO-Ph on the ITO/NiO_x_ surface. The concentration of P atoms in the ITO/NiO_x_/MeO-Ph sample is 4.45%, significantly higher than the 3.29% observed in the ITO/MeO-Ph sample, indicating that the presence of NiO_x_ enhances the adsorption of MeO-Ph.

Furthermore, the concentration of nickel (Ni) in the NiO_x_ substrate decreases after MeO-Ph adsorption, suggesting that the signal from the NiO_x_ substrate is partially obscured by the adsorbed MeO-Ph layer. To more clearly observe the differences between the samples, we performed peak fitting on the high-resolution XPS spectra of Ni 2p and O 1s. In the Ni 2p spectrum, the binding energy of Ni^3+^ increased from 855.78 eV to 855.97 eV after the adsorption of MeO-Ph, with a similar shift observed for Ni^2+^, which increased from 854.14 eV to 854.33 eV. Comparable changes were also observed in the O 1s XPS signals of NiO_x_/MeO-Ph compared to pristine NiO_x_. These chemical shifts suggest that oxygen atoms in MeO-Ph donate lone pair electrons to the vacant orbitals of nickel atoms, leading to the formation of coordination covalent bonds (P=O-Ni) at the interface. This provides compelling evidence of a charge transfer between MeO-Ph and NiO_x_ [23,24,25]. Additionally, in the O 1s spectrum, a signal corresponding to C-O at 532.25 eV was detected in the NiO_x_/MeO-Ph sample, which originates from the methoxy group of MeO-Ph. This further confirms the successful adsorption of MeO-Ph onto the NiO_x_ surface [22]. Based on previous studies [23], we propose a bonding model between NiO_x_ and MeO-Ph (Figure 1d,e). Compared to ITO, NiO_x_ prepared via ALD exhibits metal vacancies (V_Ni_) on its surface, which increase the number of highly reactive oxygen dangling bonds. This provides additional adsorption sites for MeO-Ph. This enables the formation of a robust tridentate adsorption configuration.

From the AFM images (Figure S2a–c), a noticeable increase in RMS roughness is observed for NiO_x_/MeO-Ph and MeO-Ph surfaces compared to bare NiO_x_. This indicates the successful attachment of MeO-Ph onto the substrate surface. Due to the limitations of the spin-coating process, the RMS of MeO-Ph is larger than that achieved with the ALD process. Following MeO-Ph adsorption, KPFM measurements reveal a decrease in CPD from −0.41 V to −0.50 V and −0.45 V, respectively. This significant shift in CPD serves as a reliable indicator of successful MeO-Ph adsorption. Moreover, the CPD of NiO_x_/MeO-2PACz is lower than that of NiO_x_, indicating that the adsorption of MeO-Ph leads to an increase in the *W_F_* of NiO_x_. This increase is attributed to the intrinsic dipole moment of MeO-Ph, oriented from the carbazole group to the phosphonic acid group, which generates an electric field at the surface. Additionally, since the *E_F_* of MeO-Ph is lower than that of NiO_x_, electron transfer occurs from NiO_x_ to MeO-Ph upon contact, forming a space charge region. The combined effects of the dipole layer and the space charge region contribute to the enhanced *W_F_* of NiO_x_ [26].

**Figure 2:**
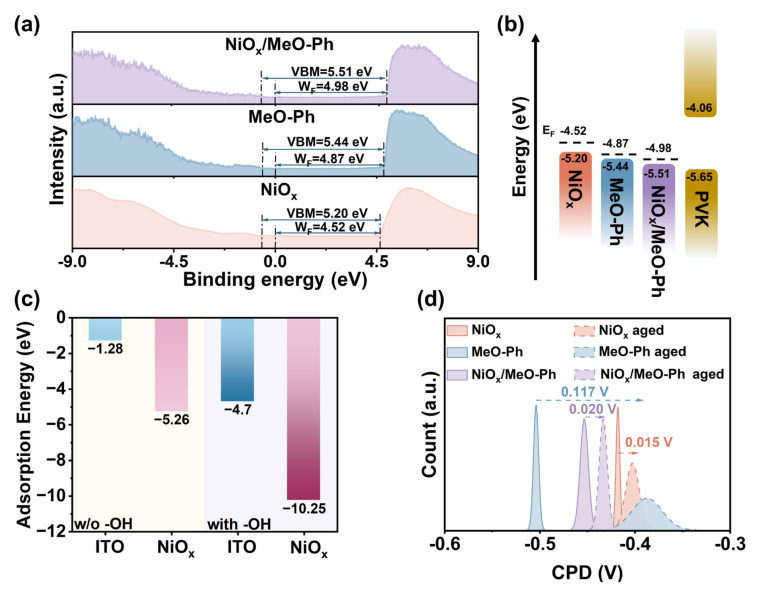
(**a**) UPS plots and (**b**) energy band schematics of NiO_x_, MeO-Ph, and NiO_x_/MeO-Ph. MeO-Ph on hydroxyl-free ITO, NiO_x_, hydroxyl-containing ITO, and NiO_x_ surfaces. (**c**) Specific adsorption energy results from theoretical calculations. (**d**) CPD changes before and after the aging of different substrates.

The *W_F_* values obtained from UPS measurements confirm that the introduction of MeO-Ph results in a more favorable alignment of the hole extraction energy levels. The *W_F_* values for NiO_x_, MeO-Ph, and NiO_x_/MeO-Ph are −4.52 eV, −4.87 eV, and −4.98 eV, respectively (Figure 2a,b). These changes are likely to contribute to improvements in *V_OC_* and *J_SC_*. Based on UV–Vis spectroscopy results, the optical band gaps (*E_g_*) for NiO_x_, MeO-Ph, and NiO_x_/MeO-Ph, calculated via Tauc plots, are 3.87 eV, 3.19 eV, and 3.82 eV, respectively. The *E_g_* value for MeO-Ph is consistent with previous literature [22]. The reduction in *E_g_* for NiO_x_/MeO-Ph is attributed to changes in the Ni^2+^/Ni^3+^ ratio [24] (Figure S3a).

We designed the corresponding structural models for DFT calculations to accurately illustrate the ability of polyhydroxy NiO_x_ to enhance the adsorption of self-assembled molecules (Figure S4). As shown in Figure 2c, when no -OH groups are present on the substrate surface, the binding energies of MeO-Ph on ITO and NiO_x_ are −1.28 eV and −5.26 eV, respectively. However, when -OH groups are present at the substrate interface, the binding energy of MeO-Ph significantly increases to −4.7 eV and −10.25 eV. These results clearly demonstrate that the presence of NiO_x_ significantly enhances the adsorption energy of MeO-Ph on the substrate, and the existence of -OH groups further promotes this increased adsorption strength. To more directly observe the stability of MeO-Ph on different substrates, we fabricated HSL structures of ITO/NiO_x_, ITO/MeO-Ph, and ITO/NiO_x_/MeO-Ph and subjected them to aging tests in air (60 °C, 50 ± 10% relative humidity). The changes in surface potential of the films before and after aging were recorded using KPFM (Oxford Jupiter XR, Oxfordshire, UK). As shown in the figure, prior to aging, all three films exhibit relatively uniform surface potential, indicating that MeO-Ph is evenly deposited on both ITO and NiO_x_ surfaces. After 480 h of aging, the CPD of ITO/MeO-Ph significantly decreases (ΔCPD ≈ 0.117 V), whereas the CPD changes of ITO/NiO_x_ and ITO/NiO_x_/MeO-Ph are only 0.015 V and 0.020 V, respectively. This suggests that the substrate with NiO_x_ as a seed layer can securely anchor MeO-Ph to the surface, thereby enhancing the operational stability of the corresponding devices. We hypothesize that the CPD change in ITO/MeO-Ph is due to the hydrolysis of the coordinating metal of the hydroxyl groups linked to the SAM molecules in the atmosphere, leading to the desorption of the SAM molecules [27,28]. Additionally, it is likely that the tridentate binding strength between SAM and NiO_x_ is stronger than the bidentate binding between SAM and ITO. Furthermore, the higher hydroxyl density on the NiO_x_ surface compared to ITO further contributes to the improved stability.

**Figure 3:**
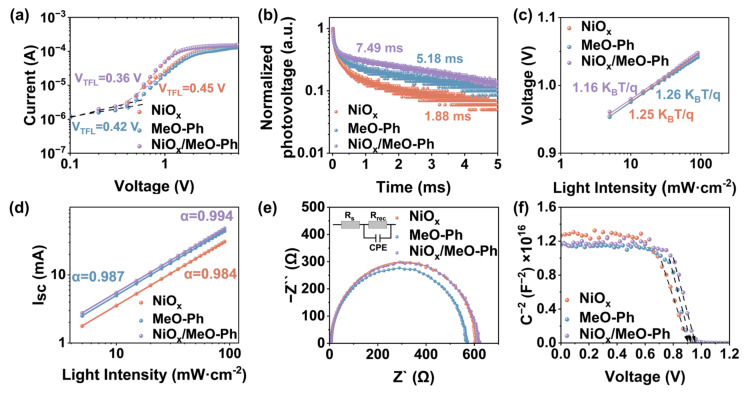
NiO_x_, MeO-Ph, and NiO_x_/MeO-Ph based on (**a**) dark *J*–*V* characteristic plots for hole-only devices; (**b**) TPV plots of NiO_x_ and MeO-Ph; (**c**,**d**) *V_OC_* and *I_SC_* plots of the corresponding PSCs as a function of light intensity; (**e**) EIS plots of the corresponding devices, the insets showing the equivalent circuits used to fit the Nyquist plots; and (**f**) Mott–Schottky plots of different PSCs.

To evaluate changes in the defect density, we fabricated p-type devices with a planar structure of ITO/HSL (NiO_x_, MeO-Ph, NiO_x_/MeO-Ph)/PVK/spiro-OMeTAD/Ag, and the *J*–*V* characteristics measured under dark conditions are shown in Figure 3a. The corresponding data are plotted in a logarithmic–logarithmic coordinate system. As the applied voltage increases, the current exhibits a linear increase in the ohmic contact region, followed by a rapid rise above the trap-filling limit voltage (*V_TFL_*). The *V_TFL_* values for NiO_x_/MeO-Ph and MeO-Ph are 0.36 V and 0.42 V, respectively, both lower than the 0.45 V for NiO_x_. Using the formula (Equation (S1)), the defect density is calculated to decrease from 8.13 × 10^−15^ cm^−3^ (NiO_x_) to 6.91 × 10^−15^ cm^−3^ (MeO-Ph) and 5.84 × 10^−15^ cm^−3^ (NiO_x_/MeO-Ph), indicating that the introduction of MeO-Ph effectively passivates the HSL/PVK interface defects [29]. To evaluate the impact of different HSL on charge extraction and transport, we performed PL spectroscopy measurements, as shown in Figure S5. Compared to the MeO-Ph/PVK sample, the NiO_x_/MeO-Ph/PVK sample exhibits a lower PL intensity, indicating that NiO_x_/MeO-Ph provides better hole extraction and transport capabilities than MeO-Ph [20]. The lowest PL intensity of NiO_x_ is likely due to carrier recombination at the NiO_x_/PVK interface defects. The TRPL was further used to quantify the carrier dynamics, where the data were fitted to a double-exponential decay function, with the results shown in Table S2. The decay time constant *τ*_1_ is attributed to the charge extraction by the HSL, while *τ*_2_ corresponds to the radiative recombination of free carriers within the perovskite layer [24]. The device based on NiO_x_/MeO-Ph exhibits the shortest *τ*_1_ and the longest *τ*_2_, confirming that the mixed HSL not only enhances the hole extraction rate but also passivates the NiO_x_/PVK interface defects.

TPV and TPC are direct methods to investigate carrier behavior (recombination or extraction) [30]. The characterization results, shown in Figure 3b and Figure S6, clearly indicate that, for devices based on NiO_x_/MeO-Ph, the photocurrent decay time is significantly reduced, while the photovoltage decay time is markedly increased. This suggests a reduction in intrinsic defects, faster charge extraction, and suppressed interfacial recombination. Additionally, to assess the charge recombination state in the devices, the dependence of *V_OC_* and *I_SC_* on light intensity was measured for the PSCs. As shown in Figure 3c, the device based on NiO_x_/MeO-Ph exhibits the smallest slope of 1.16 KB/q. The decrease in this slope can be attributed to the effective passivation of the NiO_x_/PVK interface by MeO-Ph [31]. However, we observed that the slope for devices based on MeO-Ph is slightly larger than that of NiO_x_-based devices. This may be due to the partial dissolution of the SAM during the perovskite deposition process caused by the DMF/DMSO solvents, which leads to a reduction in interface coverage and subsequent interfacial charge recombination as a result of direct contact between the perovskite and ITO [32]. Figure 3d shows the relationship between *I_SC_* and light intensity. The closer the α value is to 1, the less trap-assisted recombination occurs [33]. The results indicate that the device based on NiO_x_/MeO-Ph exhibits an α value closest to 1, which is consistent with the *V_OC_* light intensity dependence observed earlier. In EIS, the value of *R_rec_* is proportional to the diameter of the semicircle, with a larger diameter indicating less charge recombination [24]. The EIS fitting results show that the *R_rec_* values for NiO_x_, MeO-Ph, and NiO_x_/MeO-Ph are 598.1 Ω, 554.7 Ω, and 599.8 Ω, respectively. The smaller *R_rec_* value for MeO-Ph may be due to the partial dissolution of the SAM by DMF/DMSO, leading to an increase in defects at the HSL/PVK interface. The Mott–Schottky curve (Figure 3f) is typically used to measure the built-in potential (*V_bi_*) of PSCs [34]. The *V_bi_* of the NiO_x_/MeO-Ph-based device is 0.95 V, higher than that of NiO_x_ (0.92 V) and MeO-Ph (0.94 V). The higher *V_bi_* is attributed to the optimization of energy-level alignment, which provides a stronger driving force for carrier extraction and separation, aiming for a higher *V_OC_*.

**Figure 4:**
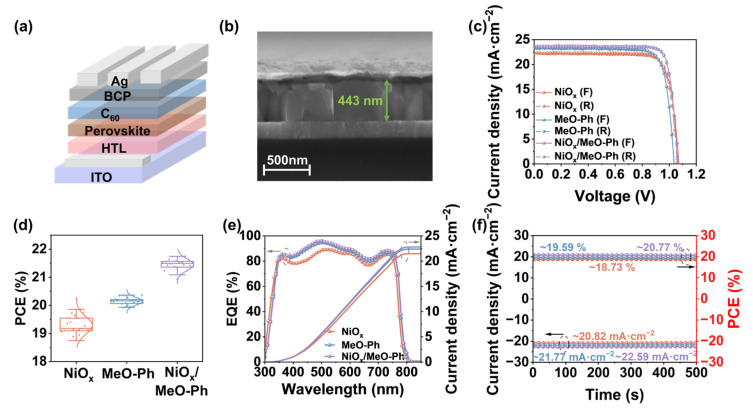
(**a**) Device schematic of p-i-n-structured PSCs. (**b**) Cross-sectional thickness plots of perovskite deposited on NiO_x_/MeO-Ph surfaces. (**c**) Forward and reverse scanning *J*–*V* curves under simulated AM 1.5 G solar illumination (100 mW cm^−2^). (**d**) Extraction of PCE distributions from *J*–*V* measurements of NiO_x_, MeO-Ph, and NiO_x_/MeO-Ph-based PSCs (20 individual devices). (**e**) EQE spectra of the best-performing device with an active area of 0.1 cm^2^ for the integrated *J_SC_*, and (**f**) power output stabilized at the point of maximum power over 500 s (SPO).

The crystallization and film formation of the perovskite layer are largely influenced by the HSL. According to the SEM images (Figure 4b and Figures S7 and S8), the perovskite grains based on MeO-Ph and NiO_x_/MeO-Ph are significantly larger, with an increase in film thickness as well. To explain these observations, we conducted water contact angle measurements (Figure S9). The results show that NiO_x_ exhibits good surface wettability, which facilitates the formation of smaller perovskite grains. In contrast, the water contact angles for MeO-Ph and MeO-Ph/NiO_x_ are significantly higher. Therefore, the changes in perovskite grain size can be attributed to an increase in the surface tension of the substrate, which enhances its hydrophobicity and reduces the mobility of grain boundaries, thereby favoring the growth of columnar grains [35]. The variation in thickness may be due to the introduction of MeO-Ph, which reduces the surface defects of NiO_x_ and provides a more favorable environment for perovskite film growth [36]. XRD patterns (Figure S10) and Tauc plots (Figure S11) show that there are no significant changes in the crystallization quality, crystal orientation, or optical bandgap of perovskite films grown on different HSL.

To evaluate the performance of NiO_x_/MeO-Ph-mixed HSL in single-junction solar cells, we fabricated p-i-n-structured PSCs with the architecture of ITO/HSL/PVK/C60/BCP/Ag, as shown in Figure 4a. Devices based on NiO_x_ and MeO-Ph as the HSL were prepared for comparison. The *J*–*V* characteristics of the optimized devices with different HSLs are shown in Figure 4b. Table S3 summarizes the photovoltaic parameters. The best-performing device with the NiO_x_/MeO-Ph-mixed HSL achieved a PCE of 21.74%, with *V_OC_*, *J_SC_*, and *FF* values of 1.07 V, 23.74 mA cm^−2^, and 84.43%, respectively. Compared to the pure NiO_x_ device, the incorporation of MeO-Ph effectively passivated interface defects, leading to significant improvements in *J_SC_*, and *FF*.

In contrast to the pure MeO-Ph device, the NiO_x_/MeO-Ph device exhibited enhanced *V_OC_*, *FF*, and *J_SC_* due to stronger SAM adsorption, reduced shunt paths, and improved hole extraction efficiency. Figure 4d and Figure S12 present the statistical distribution of various parameters for devices fabricated with different HSLs. Devices based on the NiO_x_/MeO-Ph-mixed HSL exhibit improvements in all the parameters. Among them, MeO-Ph shows the lowest *V_OC_*, which is attributed to the perovskite deposition process, where some SAM molecules are washed away, leading to significant interface carrier recombination [32], consistent with the results from EIS and *V_OC_* light intensity dependence tests. The *J_SC_* value of the devices is in good agreement with the *J_SC_* calculated from the external quantum efficiency (EQE) measurements, with the improvement in *J_SC_* attributed to the enhanced hole transport capability of the HSL. Additionally, the devices with different HSL demonstrate stable photocurrent and PCE output at the maximum power point (Figure 4f).

The detrimental redox reactions at the NiO_x_/PVK interface represent a major challenge for the commercialization of PSCs. We aim to mitigate this issue by introducing MeO-Ph. Perovskite films were deposited on different HSLs, and their changes were monitored in an atmosphere at 60 °C and 60 ± 10% relative humidity. As shown in Figure S13, after 240 h of heating, the (100) crystallographic peak of the perovskite film deposited on the three different HSLs still predominated. To gain further insight into whether the adverse interfacial reactions were suppressed, we calculated the intensity ratio of PbI_2_(001) to PVK(100) over time. The results indicated that, for perovskites grown on MeO-Ph and NiO_x_/MeO-Ph, the PbI_2_(001)/PVK(100) ratio was significantly lower compared to those grown on NiO_x_. This phenomenon suggests that the introduction of MeO-Ph effectively alleviates the detrimental redox reactions at the NiO_x_/PVK interface.

**Figure 5:**
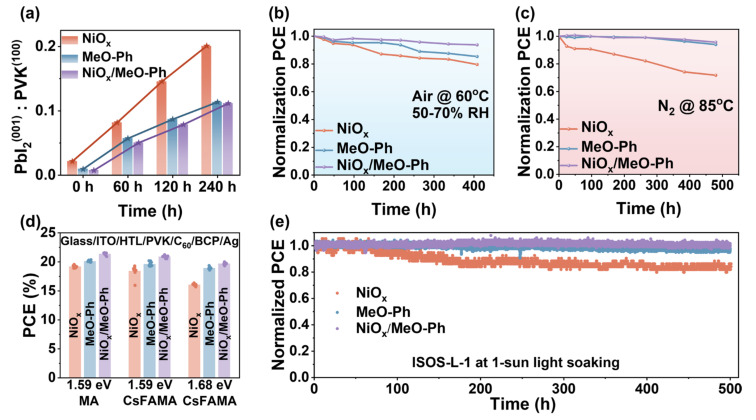
(**a**) Plots of normalized PbI_2_(001):PVK(100) intensities vs. aging time for stability measurements were performed at 60 °C and 60 ± 10% relative humidity. Long-term stability tests of unencapsulated PSCs in (**b**) air at 60 ± 10% relative humidity and 60 °C. and (**c**) an N_2_ environment at 85 °C. (**d**) PCE performances of NiO_x_, MeO-Ph, and NiO_x_/MeO-Ph in different perovskites. (**e**) Stabilized power output at the MPP at RT and N_2_ under 1 sun illumination.

To further demonstrate the advantages of the NiO_x_/MeO-Ph hybrid HSL, we investigated the operational stability of the corresponding devices under different environmental conditions. The results reveal that the hybrid HSL-based devices exhibit the highest stability, whether in an ambient atmosphere at 60 °C with a relative humidity of 60 ± 10% (Figure 5b) or in a nitrogen environment at 85 °C (Figure 5c). The efficiency degradation observed for the pure MeO-Ph device may be attributed to desorption or detachment of the SAM under high-humidity conditions [27], as no significant efficiency loss was observed in the nitrogen environment at 85 °C. To verify the applicability of the hybrid HSL, we fabricated devices using various perovskite compositions, including MAPbI_3_ (1.59 eV), Cs_0.05_(FA_0.92_MA_0.08_)_0.95_Pb(I_0.92_Br_0.08_)_3_ (1.59 eV), and Cs_0.05_FA_0.8_MA_0.15_Pb(I_0.75_Br_0.25_)_3_ (1.68 eV), and analyzed the variations in *J_SC_* and PCE. As shown in Figure 5d and Figure S14, the hybrid HSL consistently delivers significant enhancements in both *J_SC_* and PCE compared to NiO_x_ and MeO-Ph, regardless of the same bandgap with different compositions or different bandgaps. This confirms that the hybrid HSL possesses broad applicability across various perovskite systems. Moreover, the MPP tracking under harsh conditions (unencapsulated, continuous illumination (1 sun), and negative electric bias) (Figure 5e) showed that the MPP of NiO_x_-based PSCs dropped quickly (~79% after 500 h), while the PSCs with NiO_x_/MeO-Ph as the HSL maintain over 95% of its initial PCE value after 500 h, indicating better operational stability.

## 3. Materials and Methods

### 3.1. Materials and Synthesis

All reagents and solvents used were commercially available without further purification. Methylammonium iodide (MAI) was synthesized by the reaction of hydriodic acid (57 wt% in H_2_O, Aldrich, St. Louis, MO, USA) and methylamine (30–33 wt% in ethanol, Aladdin, Shanghai, China). Formamidinium iodide (FAI), methylammonium bromide (MABr), and methylammonium chloride (MACl) were synthesized according to the procedures in previous publications [22]. Lead iodide (PbI_2_), lead bromide (PbBr_2_), cesium iodide (99.9%), N,N-dimethylformamide (DMF, 99.8%, anhydrous), dimethyl sulfoxide (DMSO, 99.9%, anhydrous), N-methylpyrrolidone (NMP), chlorobenzene (CB, 99.8%, anhydrous), ethyl acetate (EA, 99.8%, anhydrous), 2-propanol (IPA, 99.8%, anhydrous), and nickel oxide (Ni_2_O_3_) were purchased from Sigma-Aldrich. Lead iodide (99.998%) was purchased from TCI. Fullerene-C60 and 2,9-dimethyl-4,7-diphenyl-1,10-phenanthroline (BCP) were purchased from Lumtec (Taiwan, China). Bis-methylcyclopentadienyl-nickel (Ni(MeCp)_2_, 99.9999%) was purchased from Nanjing Ai Mou Yuan Scientific Equipment (Nanjing, China). The synthesis procedure of MeO-PhPACz was reported in a previous work [22].

### 3.2. Computational Method

All the calculations based on the density functional theory (DFT) and the Projector Augmented Wave (PAW) potential were implemented with Device Studio (https://iresearch.net.cn/cloudSoftware, accessed on 22 March 2024, 2023A), which provides a number of functions for performing visualization, modeling, and simulation. The molecular geometry simulation used BDF [37,38,39,40] software (2024A) integrated in the Device Studio program. We used the B3LYP functional to calculate the molecules and optimized the molecular structure using the Def2-SVP basis set. For calculation of the doping mechanism, DFT calculations were performed by using the DS-PAW [41] package (2024A) in the Device Studio program. The generalized gradient approximation in the Perdew–Burke–Ernzerhof (PBE) format was used to compute exchange and correlation energies, and a plane wave basis set cutoff energy of 650 eV was adopted. Grimme’s DFT-D3 was used for dispersion correction. A mesh of 1 × 1 × 1 gamma-centered k-points was used for the Brillouin zone integration. The criterion of the electron self-consisting iteration was set as 1.0 × 10^−5^ eV, and the maximum force was relaxed down to 0.05 eV Å^−1^.

### 3.3. Device Fabrication

#### 3.3.1. Preparation of the Hole Selective Layer

The preparation of ALD NiO_x_:indium tin oxide (ITO) glass (7–9 Ω m^−2^ square, AGC, Tokyo, Japan) was ultrasonically cleaned in deionized water and ethanol for 15 min each time and dried with N_2_ flow. High-temperature tape was applied to one side of the ITO to prevent back contact from being formed by NiO_x_ deposited in this area. Next, the substrates were further cleaned with O_2_ plasma for 10 min. The ITO was transferred to the ALD reaction chamber (PEALD-200R, Kemin Electronic Equipment Technology Co., Jiaxing, China) with the substrate temperature set at 150 °C, chamber pressure below 10^−3^ torr, and reaction gas flow rate of 50 sccm. ALD NiO_x_ was deposited using Ni(MeCp)_2_ as the precursor and O_2_ plasma as the co-reactant. The Ni(MeCp)_2_ bubbler was kept at 60 °C to ensure adequate vapor pressure and dosed using Ar carrier gas through a delivery line heated to 100 °C. Each ALD cycle consisted of 0.9 s Ni(MeCp)_2_ dose, 10 s purge time, 7.5 s O_2_ plasma exposure (200 W), and 10 s purge time. After deposition, the NiO_x_ was quickly transferred to an N_2_ glove box, and the high-temperature tape was peeled off before proceeding to the next application.

MeO-Ph hole selective layer: The cleaned ITO substrates were treated with O_2_ plasma for further cleaning. A 2 mg/mL solution of MeO-PhPACz in ethanol was spin-coated onto the substrate at 3000 rpm for 30 s, followed by annealing on a hot plate at 100 °C.

NiO_x_/MeO-Ph hybrid hole selective layer: A 2 mg/mL solution of MeO-PhPACz in ethanol was spin-coated onto the ALD-deposited NiO_x_ at 3000 rpm for 30 s, followed by annealing on a hot plate at 100 °C.

#### 3.3.2. Fabrication Process of Perovskite Films

MAPbI_3_ perovskite films: The 1.3 M perovskite precursor solution was prepared by dissolving a mixture of MAI and PbI_2_ in a mixed solvent DMF/DMSO/NMP (835:75:90, *v*/*v*/*v*) and then magnetically stirring for several hours. Then, 60 μL solutions were spin-coated at 1000 rpm for 10 s and 5000 rpm for 20 s, and 140 μL of CB was dropped on the substrates in the last 5 s to form intermediate films. The resulting perovskite film was annealed at 100 °C for 10 min.

Cs_0.05_(FA_0.92_MA_0.08_)_0.95_Pb(I_0.92_Br_0.08_)_3_ perovskite films: The 1.47 M perovskite precursor solution was prepared by dissolving a mixture of PbI_2_, CsI, PbBr_2_, MABr, and FAI in a mixed solvent DMF/DMSO (4:1, *v*/*v*) and then magnetically stirring for several hours. Then, 60 μL solutions were spin-coated at 1000 rpm for 10 s and 5000 rpm for 25 s, and 110 μL of EA was dropped on the substrates in the last 10 s to form intermediate films. The resulting perovskite film was annealed at 100 °C for 20 min.

Cs_0.05_FA_0.8_MA_0.15_Pb(I_0.75_Br_0.25_)_3_ perovskite films: A 1.4 M wide bandgap (1.68 eV) perovskite precursor solution was prepared by dissolving a mixture of FAI, MABr, PbI_2_, PbBr_2_, and CsI in a mixed solvent DMF/DMSO (4:1, *v*/*v*) and then magnetically stirring for several hours. Then, 100 μL perovskite precursor solution was spread on the whole area of the HSLs/ITO substrates and spun using the spin-coating process (3500 rpm for 40 s, 5 s acceleration). According to the antisolvent method, 150 μL of ethyl acetate was dropped on the film in 10 s before the end of the program. The resulting perovskite film was then annealed at 100 °C for 30 min.

Finally, 25 nm of C60 was sequentially evaporated at rates of 0.2 Å/s. Next, 140 μL of BCP solution (0.5 mg/mL in IPA) was dynamically spin-coated at 6000 rpm for 30 s. Finally, a 120 nm Ag electrode was thermally evaporated under a vacuum of 5 × 10^−4^ Pa.

### 3.4. Characterizations and Measurements

The grazing incidence X-ray diffraction (GI-XRD) measured by X-ray diffraction (XRD, Rigaku TTRAXIII, Mito, Japan) at an incident angle of 0.5°. X-ray diffraction (XRD) was measured with a PANalytical X’Pert3 powder diffractometer (Malvern Panalytical, Shanghai, China) equipped with a Cu-sealed tube (λ = 1.541874 Å) at 40 kV and 40 mA. Ultraviolet–Visible (UV–Vis) absorption spectra and transmission spectra were recorded on a Cary 5000 spectrophotometer (Agilent, Santa Clara, CA, USA) The water contact angle was measured by a DataPhysics contact angle tester (DataPhysics, Filderstadt, Germany). The top view and cross-section morphologies of the perovskite films were investigated using field emission scanning electron microscopy (SEM) (Apreo S LoVac, Thermo, Waltham, MA, USA). The surface roughness and surface potential of perovskite films by atom force microscopy (AFM) and kelvin probe force microscopy (KPFM) were carried out using Oxford Jupiter XR (Oxfordshire, UK). The X-ray photoelectron spectrum (XPS) and ultraviolet photoelectron spectroscopy (UPS) were performed using an X-ray photoelectron spectroscopy system (Axis Supra, Shimadzu, Kyoto, Japan) with Al Kα X-ray radiation (1486.6 eV) as the X-ray source. Photoluminescence (PL) and time-resolved photoluminescence (TRPL) were carried out with a series of fluorescence spectrometers (FLS-980). The excitation and emission wavelengths were 460 nm and 770 nm for the TRPL measurements, respectively. The transient photovoltage (TPV), transient photocurrent (TPC), electrochemical impedance spectroscopy (EIS), and Mott–Schottky measurements were carried out using an electrochemical workstation (Zennium Zahner, Kronach, Germany). Space-charge limited current (SCLC) measurement was performed on a Keithley 2401 source meter ranging from 0 V to 5 V. The photovoltaic performance was measured on a source meter (Keithley 2420, Cleveland, OH, USA) under AM 1.5 G (0.1 W/cm^2^) illumination with a solar simulator (Enli Tech, Taiwan, China); the current density–voltage (*J–V*) characteristics were recorded by a source meter (Keithley 2420) with 100 mV/s scan speed. The external quantum efficiency (EQE) spectrum was measured by the EQE system (QE-R 3011, Enli Tech, Taiwan, China) in DC mode.

### 3.5. Stability Measurement

The long-term stability assessment of the perovskite solar cells was carried out by repeating the *J*–*V* characterizations over various times. For the thermal stability test, the devices were stored at 85 °C in N_2_ for 500 h, then 60 °C and 60% RH in the air for 400 h. For the storage stability test, unencapsulated devices were kept in dark conditions with N_2_ at room temperature for 960 h. Thereafter, they were placed in air at 25 °C with a relative humidity of 60% for 120 h. The long-term MPP tracking was carried out on the Multi-Channels Solar Cells Stability Test System (Wuhan 91PVKSolar Technology Co. Ltd., Wuhan, China). The solar cells were fixed at the *V_mpp_* and under constant 1 sun illumination, and the current density variation was recorded every 10 s at room temperature in a nitrogen-flowing atmosphere.

## 4. Conclusions

In summary, the NiO_x_/MeO-Ph hybrid HSL strategy offers multiple advantages. On the one hand, compared to pure MeO-Ph, the incorporation of NiO_x_ provides more adsorption sites, enhances the adsorption strength, facilitates energy band alignment at the HSL/PVK interface, prevents direct contact between the perovskite and ITO, and improves the hole selectivity of the HSL. On the other hand, compared to pure NiO_x_, the introduction of MeO-Ph achieves dual passivation of defects at the perovskite bottom surface and the HSL/PVK interface, effectively suppressing undesirable interfacial redox reactions. Devices based on the hybrid HSL exhibit superior performance and demonstrate the longest operational lifetimes under various environmental conditions. These findings confirm that the application of the hybrid HSL in single-junction PSCs is a highly advantageous approach. We believe this study provides a solid foundation for the development of high-performance and highly stable p-i-n PSCs and accelerates their path toward commercialization.

## Data Availability

All data are available upon request.

## Supplementary Materials

The following supporting information can be downloaded at https://www.mdpi.com/article/10.3390/molecules30061299/s1: Figure S1: Light transmittance of NiO_x_, MeO-Ph, and NiO_x_ /MeO-Ph; Figure S2: Surface roughness of (a) NiO_x_, (b) MeO-Ph, and (c) NiO_x_/MeO-Ph. CPD distribution of (d,e) three films (from left to right: NiO_x_, MeO-Ph, and NiO_x_/MeO-Ph); Figure S3: (a,b) (αhv)x vs. hv plots (x = 2 or 1/2) of NiO_x_, MeO-Ph, and NiO_x_/MeO-Ph; Figure S4: Theoretical adsorption mode of MeO-Ph on surfaces without -OH (a) ITO and (b) NiO_x_, and with -OH (c) ITO and (d) NiO_x_; Figure S5: NiO_x_, MeO-Ph, and NiO_x_/MeO-Ph based on (a) PL spectra and (b) TRPL spectra; Figure S6: TPC plots of NiO_x_, MeO-Ph, and NiO_x_/MeO-Ph-based PSCs; Figure S7: Top view particle size distribution plots of perovskite deposited on (a) NiO_x_, (b) MeO-Ph, and (c) NiO_x_/MeO-Ph surfaces; Figure S8: Cross-sectional thickness plots of perovskite deposited on (a) NiO_x_ and (b) MeO-Ph surfaces; Figure S9: Water contact angle plots of perovskite deposited on (a) NiO_x_, (b) MeO-Ph, and (c) NiO_x_/MeO-Ph surfaces; Figure S10: XRD spectra plots of perovskite deposited on NiO_x_, MeO-Ph, and NiO_x_/MeO-Ph surfaces; Figure S11: (αhν)^2^ vs. energy (eV) plots of perovskite deposited on NiO_x_, MeO-Ph, and NiO_x_/MeO-Ph surfaces; Figure S12: Extraction of (a) *V_OC_*, (b) *J_SC_*, and (c) *FF* distributions from *J*–*V* measurements of NiO_x_, MeO-Ph, and NiO_x_/MeO-Ph-based PSCs (20 individual devices); Figure S13: Stability measurements were performed at 60 °C and 60 ± 10% relative humidity. Includes the evolution of XRD patterns of perovskite deposited on (a) NiO_x_, (b) MeO-Ph, and (c) NiO_x_/MeO-Ph; Figure S14: *J_SC_* performances of NiO_x_, MeO-Ph, and NiO_x_/MeO-Ph in different perovskites; Figure S15: (a) *J_SC_* and (b) PCE performances of NiO_x_/(MeO-2PACz or MeO-Ph)-mixed hole transport layers; Table S1: Atomic concentrations of various major elements of NiO_x_, MeO-Ph, and NiO_x_/MeO-Ph; Table S2: TRPL fitting parameters based on NiO_x_, MeO-Ph, and NiO_x_/MeO-Ph devices; Table S3: NiO_x_, MeO-Ph, and NiO_x_/MeO-Ph as the photovoltaic parameters for the best HSL PSCs.
