# Supplementary material for: ALD-Deposited Hydroxyl-Rich NiOx to Enhance SAM Anchoring for Stable and Efficient Perovskite Solar Cells
Source: Molecules. 2025 Mar 13;30(6):1299. doi: 10.3390/molecules30061299 (PMC11946364; doi:10.3390/molecules30061299)
Supplement: Supplementary file 1 [file molecules-30-01299-s001.zip › molecules-3512107-supplementary.pdf]

Supporting information

ALD Deposited Hydroxyl-Rich NiO_x Enhances SAM Anchoring for Stable and Efficient Perovskite Solar Cells

Fengming Guo [1,2,3,4], Xuteng Yu [2,3,5], Yuheng Li [1,2,3,4], Yong Chen [2,3,4], Chi Li [2,3,5], Chuming Liu [2,3,5] and Peng Gao [2,3,5,*]

[1] College of Chemistry and Materials Science, Fujian Normal University, Fuzhou, Fujian 350108, China
[2] State Key Laboratory of Structural Chemistry, Fujian Institute of Research on the Structure of Matter, Chinese Academy of Sciences, Fuzhou, Fujian 350002, P. R. China
[3] Laboratory for Advanced Functional Materials, Xiamen Institute of Rare Earth Materials, Haixi Institute, Chinese Academy of Sciences, Xiamen 361021, China
[4] Fujian College, University of Chinese Academy of Sciences, Fuzhou, 350002, China
[5] University of Chinese Academy of Sciences, Beijing 100049, China
* Correspondence: peng.gao@fjirsm.ac.cn

Supplementary Note 1. n_{trap} calculation.

The trap density (n_{trap}) is determined by the trap-filled limit voltage (V_{TFL}) based on the following equation[1]:

$$n_{trap} = \frac{2V_{TFL}}{qL^2}\varepsilon_r\varepsilon_0 \qquad \text{(S-eq 1)}$$

Where L is the thickness of the perovskite film, V_{TFL} is the onset voltage of the trap-filled limit region, ε_r is the relative dielectric constant of perovskite film, ε_0 is the vacuum permittivity, and q is the electron charge.

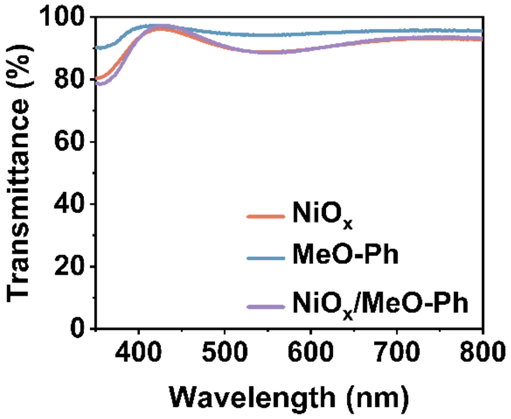

Figure S1. Light transmittance of NiO_x, MeO-Ph, and NiO_x/MeO-Ph

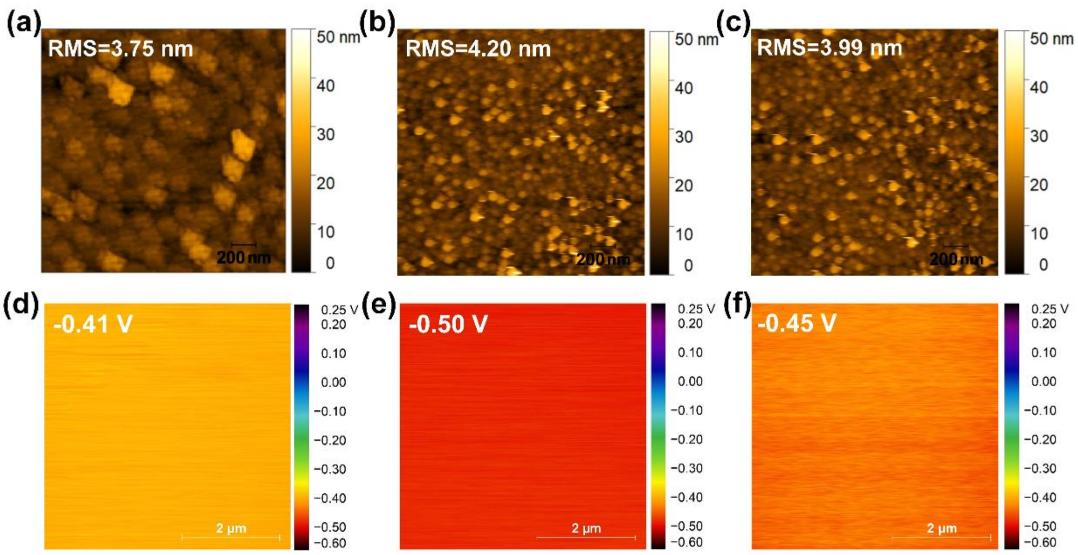

Figure S2. Surface roughness of (a) NiO_x, (b) MeO-Ph and (c) NiO_x/MeO-Ph, CPD distribution of (d-e) three films (from left to right: NiO_x, MeO-Ph, NiO_x/MeO-Ph).

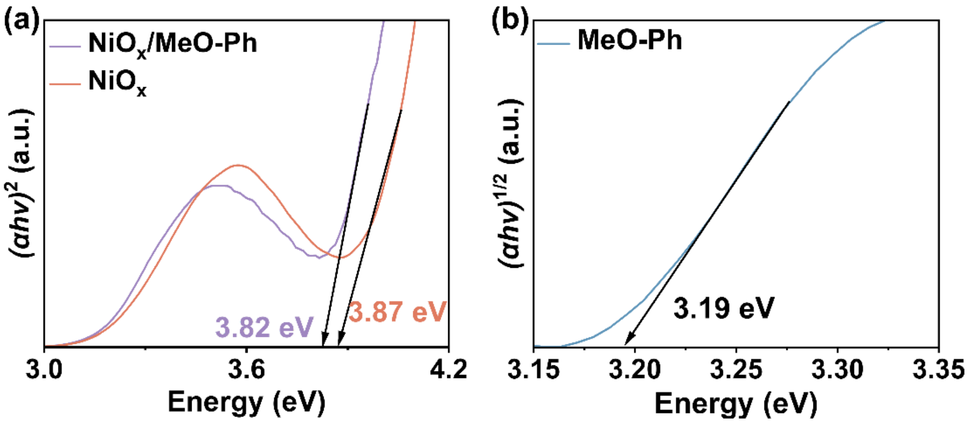

Figure S3. (a-b) $(\alpha hv)^x$ vs. hv plots (x = 2 or 1/2) of NiO_x, MeO-Ph, and NiO_x/MeO-Ph.

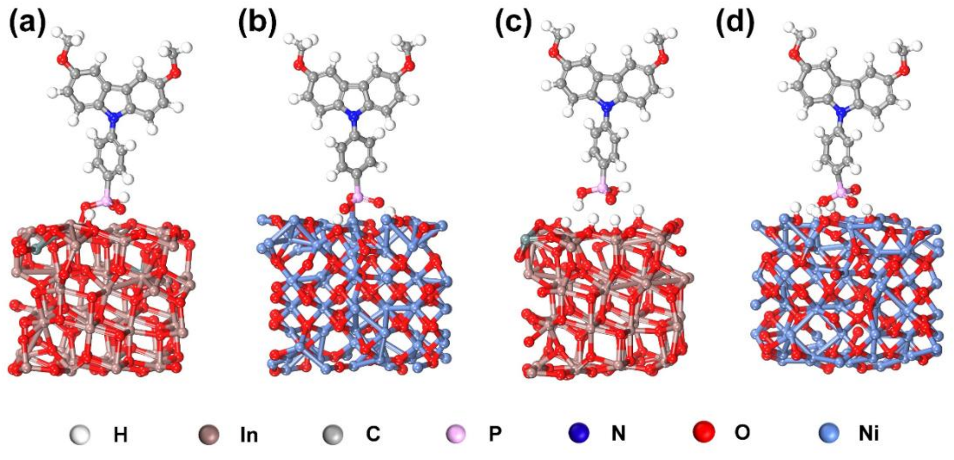

Figure S4. Theoretical adsorption mode of MeO-Ph on surfaces without -OH (a) ITO, (b) NiO_x, and with -OH (c) ITO, (d) NiO_x.

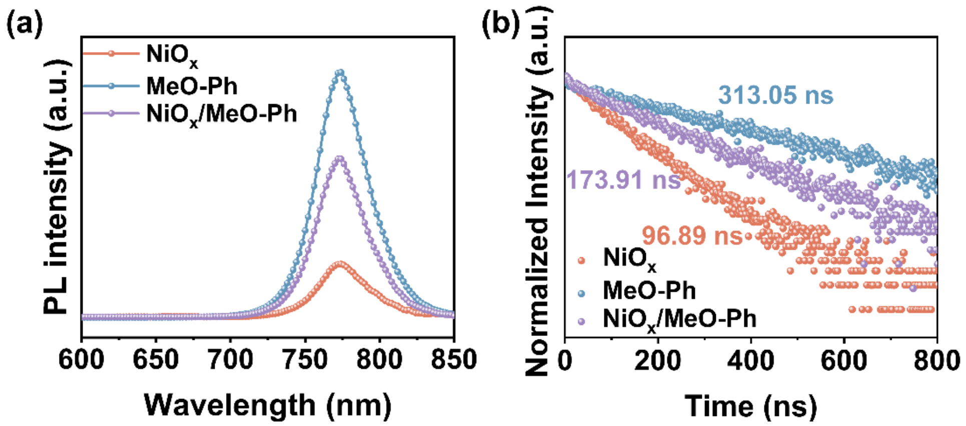

Figure S5. NiO_x, MeO-Ph, and NiO_x/MeO-Ph based on (a) PL spectra, and (b) TRPL spectra.

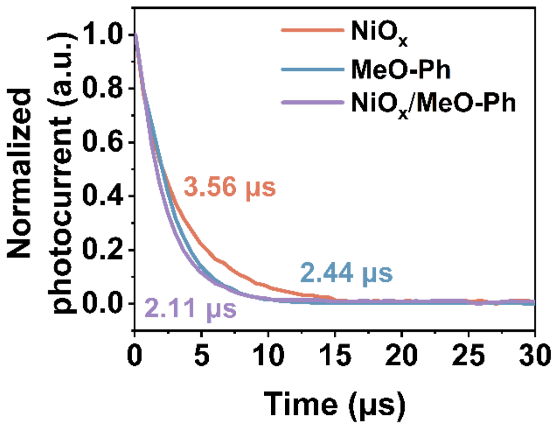

Figure S6. TPC plots of NiO_x, MeO-Ph, and NiO_x/MeO-Ph based PSCs.

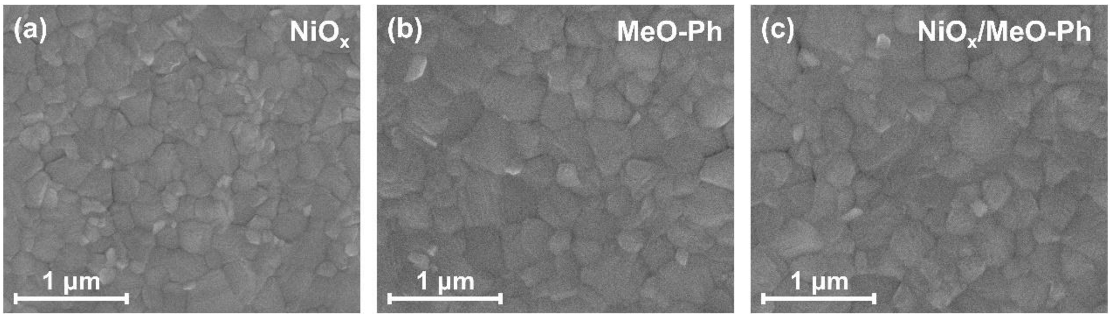

Figure S7. Top-view particle size distribution plots of perovskite deposited on (a) NiO_x, (b) MeO-Ph and (c) NiO_x/MeO-Ph surfaces.

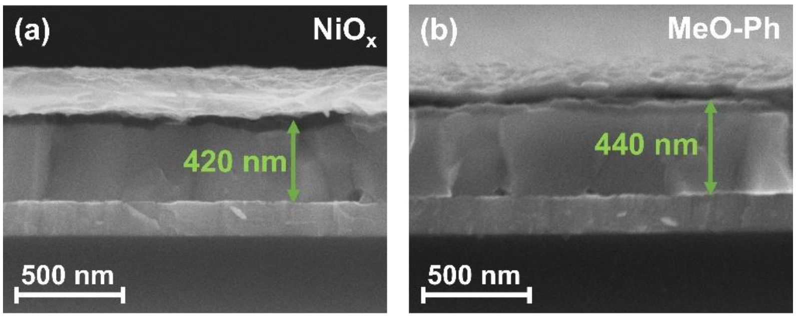

Figure S8. Cross-sectional thickness plots of perovskite deposited on (a) NiO_x and (b) MeO-Ph surfaces.

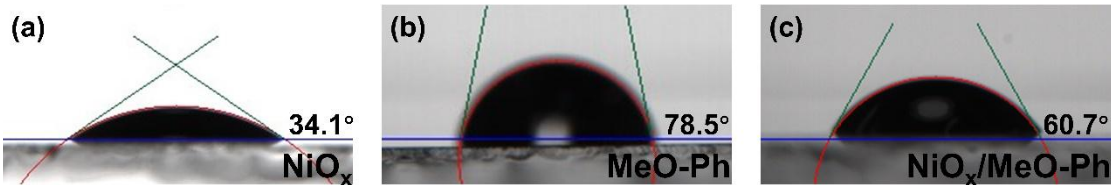

Figure S9. Water contact angle plots of perovskite deposited on (a) NiO_x, (b) MeO-Ph and (c) NiO_x/MeO-Ph surfaces.

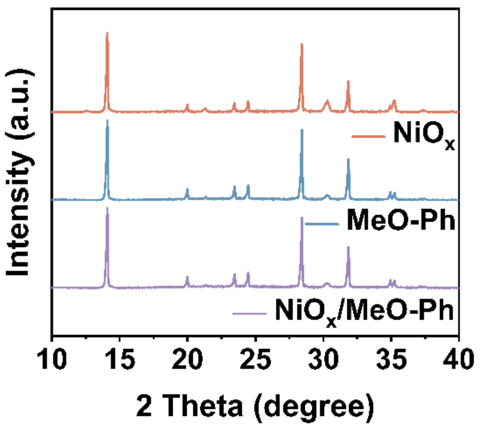

Figure S10. XRD spectra plots of perovskite deposited on NiO_x, MeO-Ph and NiO_x/MeO-Ph surfaces.

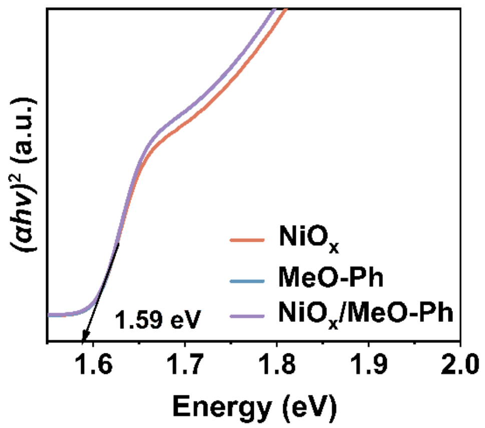

Figure S11. $(\alpha hv)^2$ vs. Energy (eV) plots of perovskite deposited on NiO_x, MeO-Ph and NiO_x/MeO-Ph surfaces.

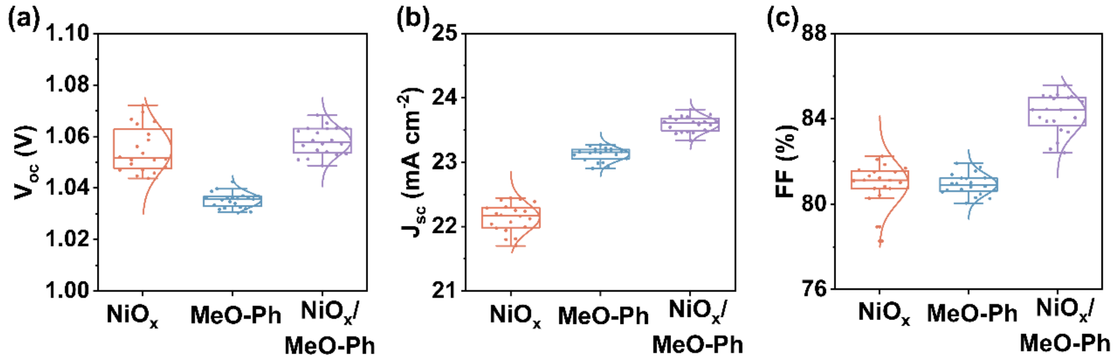

Figure S12. Extraction of (a) V_{OC}, (b) J_{SC} and (c) FF distributions from J-V measurements of NiO_x, MeO-Ph, and NiO_x/MeO-Ph based PSCs (20 individual devices).

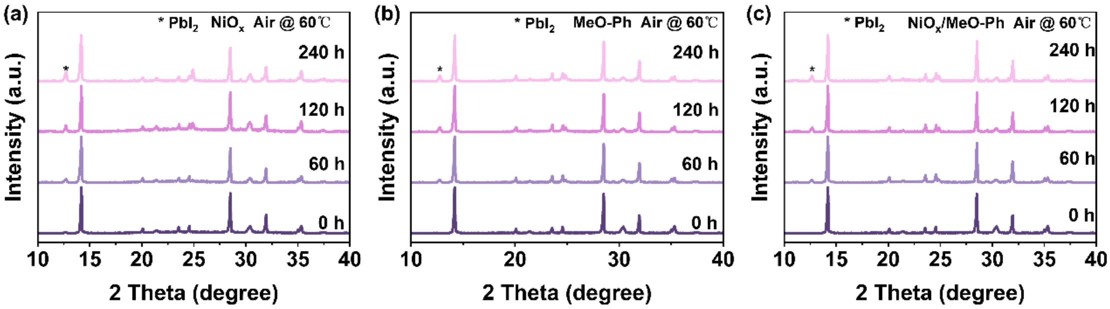

Figure S13. Stability measurements were performed at 60 °C and 60 ± 10% relative humidity. Includes evolution of XRD patterns of perovskite deposited on (a) NiO_x, (b) MeO-Ph and (c) NiO_x/MeO-Ph.

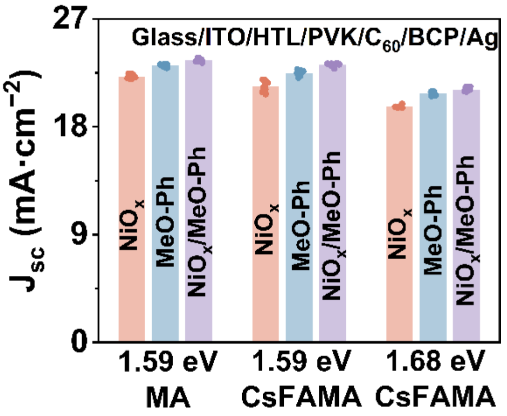

Figure S14. J_{SC} performances of NiO_x, MeO-Ph, and NiO_x/MeO-Ph in different perovskite.

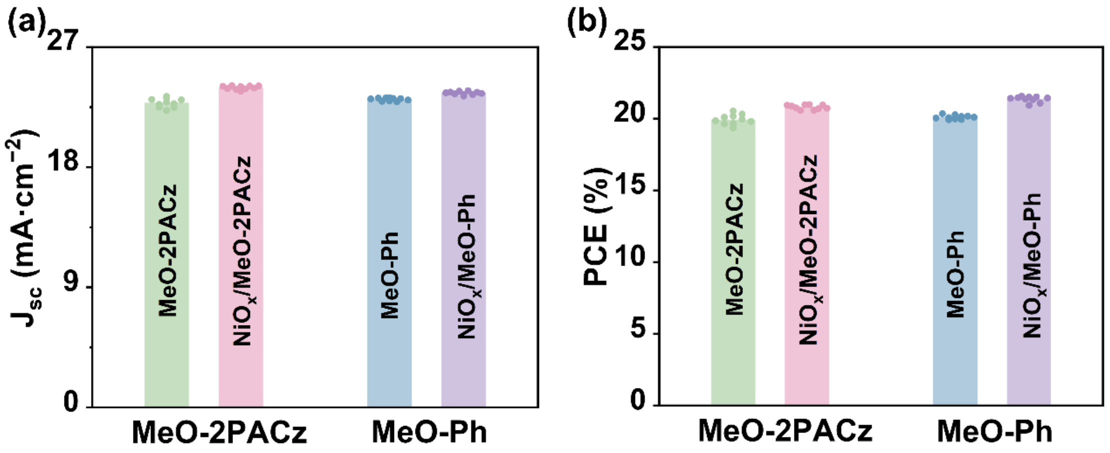

Figure S15. (a) J_{SC} and (b) PCE performances of NiO_x/(MeO-2PACz or MeO-Ph) mixed hole transport layer.

Table S1. Atomic concentrations of various major elements of NiO_x, MeO-Ph, and NiO_x/MeO-Ph

Sample	P 2p	N 1s	O 1s	C 1s	In 3d	Ni^{3+}	Ni^{2+}
ITO/NiO_x	N/A	N/A	47.94	25.98	0.59	19.41	6.08
ITO/MeO-Ph	3.29	3.32	27.97	54.22	11.19	N/A	N/A
ITO/NiO_x/MeO-Ph	4.45	3.51	25.42	59.97	0.42	4.72	1.50

Table S2. TRPL fitting parameters based on NiOx, MeO-Ph, and NiO_x/MeO-Ph devices. Where $\tau_{AVE} = (A_1\tau_1 + A_2\tau_2) / (A_1 + A_2)^2$

Sample	A_1	τ_1 (ns)	A_2	τ_2 (ns)	τ_{AVE} (ns)
NiO_x	0.17	20.66	0.79	99.27	96.89
MeO-Ph	0.11	17.53	0.75	176.14	313.05
NiO_x/ MeO-Ph	0.04	17.03	0.72	314.03	173.91

Table S3. NiO_x, MeO-Ph, and NiO_x/MeO-Ph as photovoltaic parameters for HSL best PSCs

Devices	Scan direction	J_{SC} (mA cm^{-2})	V_{OC} (V)	*FF* (%)	PCE (%)
NiO_x	Forward	22.23	1.07	80.82	19.75
	Reverse	22.31	1.07	81.86	19.86
MeO-Ph	Forward	23.27	1.04	81.01	20.29
	Reverse	23.22	1.04	81.54	20.36
NiO_x/MeO-Ph	Forward	23.47	1.06	85.12	21.64
	Reverse	23.74	1.07	84.43	21.75

Reference

1. Dong, Q.; Fang, Y.; Shao, Y.; Mulligan, P.; Qiu, J.; Cao, L.; Huang, J. Electron-Hole Diffusion Lengths > 175 Mm in Solution-Grown $CH_3NH_3PbI_3$ Single Crystals. *Science* **2015**, *347*, 967–970, doi:10.1126/science.aaa5760.
